# Construction of Osteosarcoma Diagnosis Model by Random Forest and Artificial Neural Network

**DOI:** 10.3390/jpm13030447

**Published:** 2023-02-28

**Authors:** Sheng Li, Yukang Que, Rui Yang, Peng He, Shenglin Xu, Yong Hu

**Affiliations:** Department of Orthopedics, First Affiliated Hospital of Anhui Medical University, Hefei 230022, China

**Keywords:** osteosarcoma, biomarker, random forest classifier, neural network model, gene expression Omnibus

## Abstract

Osteosarcoma accounts for 28% of primary bone malignancies in adults and up to 56% in children and adolescents (<20 years). However, early diagnosis and treatment are still inadequate, and new improvements are still needed. Missed diagnoses exist due to fewer traditional diagnostic methods, and clinical symptoms are often already present before diagnosis. This study aimed to develop novel and efficient predictive models for the diagnosis of osteosarcoma and to identify potential targets for exploring osteosarcoma markers. First, osteosarcoma and normal tissue expression microarray datasets were downloaded from the Gene Expression Omnibus (GEO). Then we screened the differentially expressed genes (DEGs) in the osteosarcoma and normal groups in the training group. Next, in order to explore the biologically relevant role of DEGs, Metascape and enrichment analyses were also performed on DEGs. The “randomForest” and “neuralnet” packages in R software were used to select representative genes and construct diagnostic models for osteosarcoma. The next step is to validate the model of the artificial neural network. Then, we performed an immune infiltration analysis by using the training set data. Finally, we constructed a prognostic model using representative genes for prognostic analysis. The copy number of osteosarcoma was also analyzed. A random forest classifier identified nine representative genes (ANK1, TGFBR3, TNFRSF21, HSPB8, ITGA7, RHD, AASS, GREM2, NFASC). HSPB8, RHD, AASS, and NFASC were genes we identified that have not been previously reported to be associated with osteosarcoma. The osteosarcoma diagnostic model we constructed has good performance with areas under the curves (AUCs) of 1 and 0.987 in the training and validation groups, respectively. This study opens new horizons for the early diagnosis of osteosarcoma and provides representative markers for the future treatment of osteosarcoma. This is the first study to pioneer the establishment of a genetic diagnosis model for osteosarcoma and advance the development of osteosarcoma diagnosis and treatment.

## 1. Introduction

Osteosarcoma (OS) was the earliest identified human cancer and is also the most common malignant bone tumor [1]. The vast majority of cases occur in pediatric patients and adolescents between the ages of 10 and 30 years, with a peak incidence during adolescence [2]. Early diagnosis of OS is challenging, and it is highly heterogeneous and complicated, making it difficult to cure. A proportion of patients with osteosarcoma already present with metastatic disease at the time of new diagnosis (10–15%), mainly pulmonary metastases [3]. The 5-year event-free survival rate was 60% for patients with radiologically confirmed nonmetastases and only 20% for patients with multiple pulmonary metastases or metastases from other sites [4]. Consequently, the diagnosis and treatment of OS at an early stage remains a compelling problem and challenge for researchers. Therefore, there is a need to identify representative markers to provide early diagnosis and effective treatment. 

In current clinical practice, the diagnosis of osteosarcoma includes clinical symptoms, physical examination, imaging, other examinations (arteriography and positron emission tomography-computed tomography), biopsy, and laboratory tests. The diagnosis of a patient with osteosarcoma should be based on a combination of clinical, imaging and pathology findings, and all three findings should be consistent; otherwise, the correctness of the diagnosis should be questioned [5]. To date, some studies have shown that the misdiagnosis and underdiagnosis of OS based on imaging and medical history alone is still high, especially in nonadolescent patients whose incidence is approximately 23–43% [6,7].

Recently, with the development of computer science and technology, mathematical science, and high-throughput sequencing technology, precision medicine has also been gradually developed [8]. Several machine learning algorithms and definitions of supervised/unsupervised learning, such as clustering algorithms, random forests and support vector machines, have been well developed and applied in the field of medical research [9]. Meanwhile, osteosarcoma-related models have been developed. Yanqi He et al. constructed a prognostic nomogram model for patients with osteosarcoma by exploiting data from the Surveillance, Epidemiology, and End Results program [10]. Le et al. used CIBERSORTx to assess the immune cell abundance of osteosarcoma in the target database and, after certain algorithms and processing procedures, successfully constructed a mathematical model for assessing osteosarcoma progression [11].

For the diagnosis of osteosarcoma, imaging and pathological examination are currently the main methods of initial diagnosis. Therefore, related diagnostic methods or models have been developed. One study proposed a deep learning model based on reading pathological sections to improve the efficiency and accuracy of osteosarcoma diagnosis [12]. Some researchers have continuously improved the computer processing algorithms of magnetic resonance imaging (MRI) images while establishing computer-aided diagnostic processing schemes for osteosarcoma; after validating and analyzing more than 70,000 MRI images of osteosarcoma from Chinese hospitals, they proposed a system model based on MRI computer-aided processing (AIMSost) [13,14,15,16]. Artificial neural networks (ANNs) are mathematical models consisting of a large number of interconnected neurons that form diverse networks according to various connectivity methods, with biological nerves as the basis [17]. However, the construction of OS diagnosis prediction models from the perspective of neural networks has not yet been studied.

**Figure 1:**
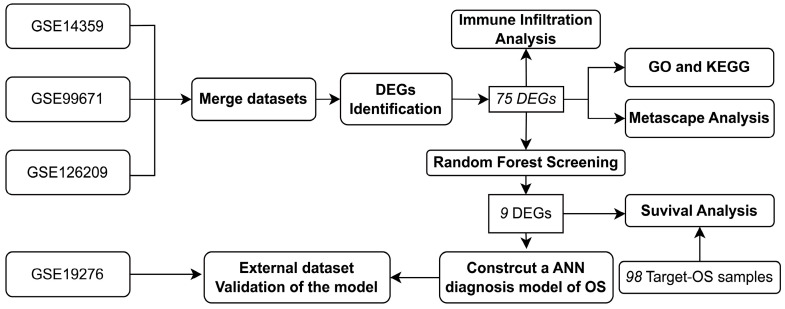
Simplified flow chart. This is the overall simplified flowchart of the whole study, which is divided into four main subsections: data collation related to diagnostic models, gene screening and analysis, diagnostic model building and validation, and diagnostic genes for prognostic correlation analysis.

As early as 1995, Baxt illustrated the great flexibility of the artificial neural network paradigm and its ability to diagnose with accuracy in a variety of fields [18]. The name and structure of artificial neural networks are inspired by the human brain and mimic early models of sensory biological neuronal signaling processing from the brain, and networks that simulate model neurons in a computer are able to “learn” to solve numerous types of problems [19]. Neural networks have been applied to many important problems in the medical field, and in some cases, they provide state-of-the-art solutions. Our study aims to create a random forest and artificial neural network diagnosis model of OS based on representative gene weights and to study the different immune cell infiltrations between OS groups and normal groups. Figure 1 shows a brief flow chart of the whole study. The model we developed can be used to distinguish between OS samples and normal samples effectively given its superiority in this study, which has extraordinary implications for the future of early diagnosis and effective treatment of OS.

## 2. Materials and Methods

### 2.1. Database from GEO and Analysis

We used Gene Expression Omnibus to obtain the gene expression datasets GSE14359, GSE99671, GSE126209, and GSE19276 (https://www.ncbi.nlm.nih.gov/geo/, access on 9 November 2022). For a more accurate study, the inclusion criteria of data in this study were as follows: primary osteosarcoma tissue and normal paraneoplastic tissue or normal bone tissue samples. In this research, GSE99671 and GSE126209 have paired datasets for each osteosarcoma patient. In the translation process of microarray probe IDs and gene symbols, when multiple probes are detected to match a gene symbol, the intermediate expression of the probe is considered to be the final level of gene expression. GSE14359, GSE99671, and GSE126209 were merged as a training group. The “ComBat” function for handling batch effects for multiple data platforms is provided using the package “sva” in R software. GSE14359 was used as a validation group. The fundamental details of the four datasets are presented in Table 1.

### 2.2. Differentially Expressed Genes and Enrichment Investigation

DEGs between the osteosarcoma and normal groups in the training group were identified by using the “limma” package in R software, and adjusted *p* values <0.05 and |log-fold change (FC)| >1 were used as significant criteria. The results were visualized by applying the “ggplot2” and “pheatmap” packages to convert the results into volcano maps and heat maps. We performed a Metascape analysis of DEGs (http://metascape.org/gp/index.html, access on 9 November 2022). In addition, we performed GO functional enrichment analysis and KEGG analysis. The genome enrichment of differentially expressed genes was determined by calling the “clusterProfiler” package in R software. In GO functional enrichment analysis, the *p*-value and q-value were set at 0.05 and 0.05, respectively. Finally, the enrichment results were presented by using the “enrichplot”, “ggplot2”, and “GOplot” packages.

### 2.3. Random Forest Analysis for DEGs and Visualization

After obtaining DEGs, we created a random forest model by using the R package “randomForest”. First, we set the optimal variable value for the binary tree node to six. The total number of trees was determined to be 500 in the random forest. The algorithm was then used to calculate the rate of mean miscalculation for all variables between the OS and normal groups. By calculating the minimum error rate of cross-validation, the best number of random forest trees can be obtained. For the next step of modeling, DEGs with significant points greater than one were selected as representative genes. The unstructured hierarchical groups of the nine representative gene training groups were reclassified, and the full results were visualized by applying the “ggplot2” and “pheatmap” packages to convert the results to heatmaps.

### 2.4. Construction of Artificial Neural Network Model

In the previous step, we obtained nine representative genes by random forest screening. The next procedure is to process the expression data of the nine representative genes to obtain the gene scores of the nine representative genes. The gene scores were obtained by analyzing their expression levels and comparing them with the median expression value of all samples. A value of zero was assigned when the gene expression level was upregulated but the representative gene expression level was low; otherwise, a value of one was assigned. When the downregulated representative gene expression level was low, a value of one was assigned; otherwise, a value of zero was assigned. Then, by going through the above steps, we end up with a sheet with nine columns representing genes and 71 rows of samples. 

The ANN model was developed by applying the R package “neuralnet”, and the model includes five hidden layers. The basic principle of the model is that the sum of each input gene score multiplied by the weight of each input gene determines the final output layer. The plane of the ROC curve was plotted using the “pROC” package in R language. The horizontal and vertical coordinates of the plane are the false positive rate and the true positive rate of the model. Additionally, the performance of the constructed model was evaluated by calculating the area under the ROC curve (AUC).

### 2.5. Validation of the Artificial Neural Network Model

In order to verify the effectiveness of the model built in this previous step, the gene expression dataset of GSE19276 was obtained from the GEO database, and gene scores were obtained in the same way by using genetic expression information. The gene scores of the validation group were input into the model, and then the scores for the predicted test group samples were obtained. Through scores for the prediction, we can obtain the grouping of the validation group predictions and then calculate the accuracy of the predictions of the osteosarcoma group and the normal group in the validation group. Then, the performance of the constructed model was also evaluated by plotting ROC curves and expressed as the AUC by using the pROC package in R 4.2.2 software.

### 2.6. Immune Infiltration Analysis

By using CIBERSORT (a gene expression profiling-based algorithm), the immune tissue cell composition can be accurately calculated. We obtained the proportion of immune cells between OS and normal individuals by using the “complot” package in R software with 1000 permutations. The results were visualized by applying the “barplot” function in R software. A *p*-value (hypothesis testing value) < 0.1 was set as the filtration condition for the correlation analysis of 22 types of immune cells, and the R package “corrplot” was used. Then, the results were visualized in a correlation heatmap. Finally, differences in the obtained immune cells between the OS and normal groups were studied by the R package “vioplot”.

### 2.7. Cox Proportional Hazards Model and Survival Analysis

We obtained RNA sequencing data and clinical information from the TARGET database for 98 patients with osteosarcoma. A proportional hazards model was constructed for these nine representative genes by the “survival” package. The model predicted each patient’s risk value and then classified them into two groups, high and low risk, based on the median risk value. Using the “pheatmap” software package, we mapped the risk profile of the patients. A survival state map and a genetic heatmap were also drawn. Then, we called the “survminer” package and performed a log-rank test to obtain the *p*-value of the difference in survival between the two groups. The hazard ratio with a 95% confidence interval was also obtained by univariate Cox regression analysis. Kaplan–Meier plots were then generated by using the “ggsurvplot” function. Finally, the ROC curves of the survival model were plotted by the survivalROC package. Copy number variant data were also obtained from TARGET for 88 patients with osteosarcoma. Online analysis was performed with GISTIC 2.0. The distribution and heatmap of osteosarcoma genomic copy number variants were plotted.

## 3. Results

### 3.1. DEG Identification

**Figure 2:**
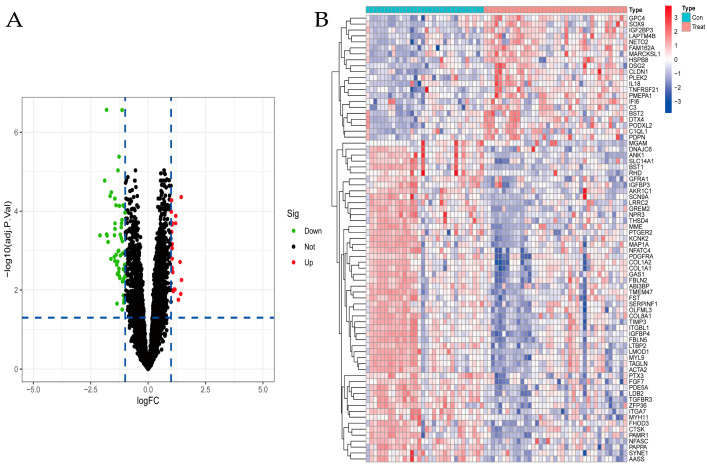
Seventy−five expression−related genes were extracted after differentially expressed gene analysis, 21 genes were upregulated, and 54 genes were downregulated in osteosarcoma tissues (Treat) compared with normal tissues (Con). (**A**) Volcano plot of DFGs between the normal and OS groups. (**B**) Heatmap of DEGs between the normal and OS groups.

The “limma” package was used to identify DEGs between the OS group and the normal group by the Bayesian test. Using *p* < 0.05 and [logFC] > 1 as significant criteria, we extracted 75 expression-related genes after differentially expressed gene analysis. Among them, 21 genes were upregulated, and 54 genes were downregulated in osteosarcoma tissues compared with normal tissues. The screened DEGs were visualized by volcano plot (Figure 2A) and heatmap (Figure 2B).

### 3.2. Metascape Analysis Resource of DEGs

**Figure 3:**
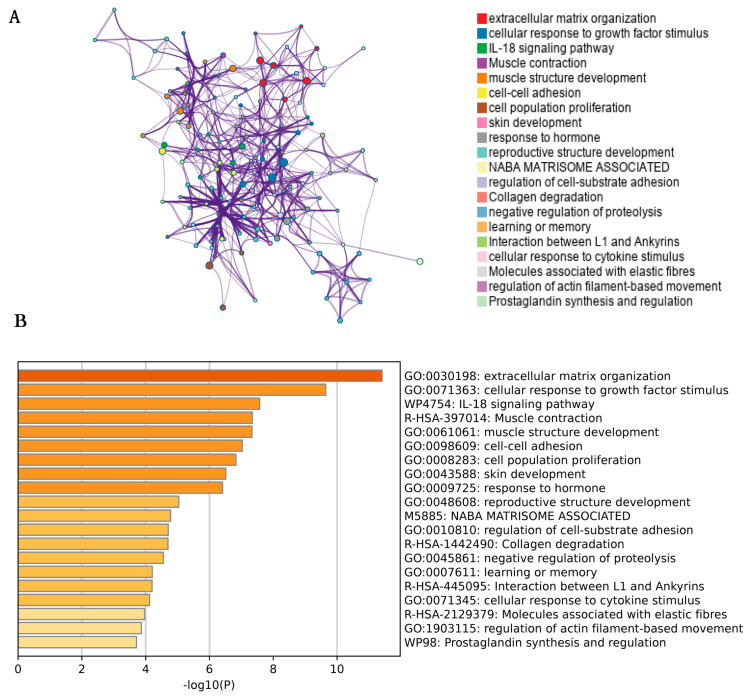
(**A**) The enrichment pathway is also presented as a network. A unique color represents each set of host factors. (**B**) The horizontal coordinate is a logarithmic value of *p* values with a base of 10 and negative values; the vertical is the different enrichment pathways that have been sorted by the value of −log10(P). The larger the −log10(P) value ranked above, the smaller the *p* values and the more significant the enrichment (and the darker the color).

Enrichment analysis of pathways and processes was performed using the following ontology resources. KEGG pathway, GO biological processes, Reactome genesets, canonical pathways, CORUM, WikiPathways and PANTHER Pathway. On the basis of member similarity, terms with *p*-value < 0.01, minimum count of 3, and enrichment factor > 1.5 were collected. We visualized the first 20 clusters in Figure 3. In the next step, a rich subset of terms with similarity > 0.3 is connected by edges to form a network graph. The terms with the best *p*-values were selected from each of the 20 clusters. We used Cytoscape5 to visualize the network, where each node represents an enriched term. Figure 3A is colored first by its cluster-ID and Figure 3B by its *p*-value.

### 3.3. Analysis of GO Enrichment and KEGG Pathways

**Figure 4:**
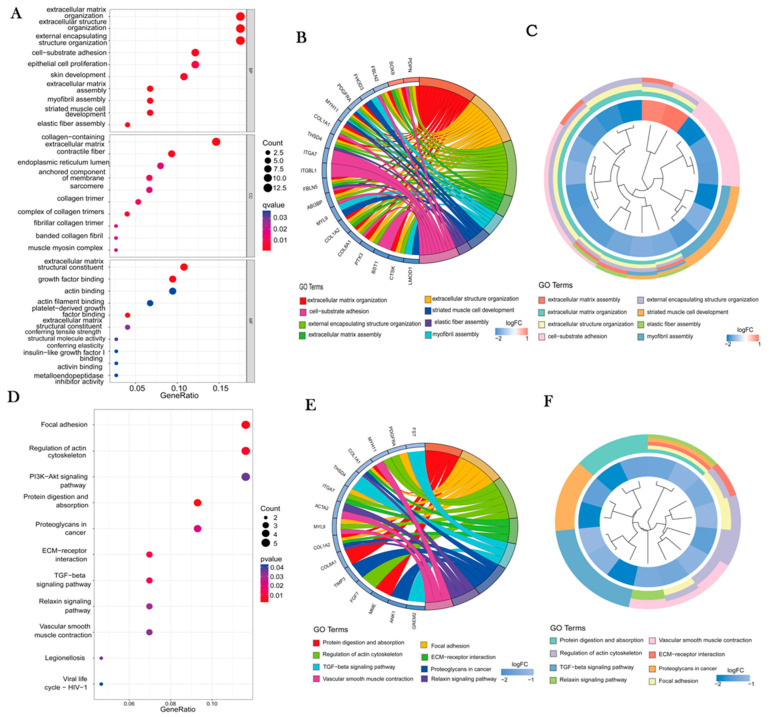
Graphs of GO and KEGG enrichment analysis results. (**A**) The results of the GO enrichment analysis of the top 10 GO terms of DFGs for BP, MF and CC are shown in three boxes with bubble plots. Gen-Ratio is on the *x*-axis, and log 10(adj P) values are on the *y*-axis. (**B/E**) GO/KEGG enrichment chord diagram. The left side indicates genes, red represents up-regulation, blue represents down-regulation, and color shades represent the number of fold changes. On the right, GO/KEGG terms are sorted by enrichment intensity change. (**C/F**) Gene clustering diagram: inner circles indicate DEGs, red circles represent up-regulated genes, blue circles indicate down-regulated genes, and the outer circle represents GO/KEGG keywords. (**D**) KEGG enrichment analysis results of DFGs. The Rich Factor (the ratio of differential genes to the total number of classified genes) was used as the *x*-axis, and the *y*-axis was the information of each enriched classification, with the size of bubbles indicating the number of differential genes and the color representing the corresponding significance information.

From the results of GO enrichment analysis, we found that DEGs were related to cellular processes and developmental processes, including cellular component organization, myofibril assembly, striated muscle development, cell-substrate adhesion, and epithelial cell proliferation (Figure 4A–C). KEGG pathway enrichment showed that DEGs were mainly enriched in tumor cellular processes, signal transduction, and organismal systems. Additionally, focal adhesion, regulation of actin cytoskeleton, and ECM-receptor interaction were identified. The PI3K-Akt signaling pathway, TGF-beta signaling pathway, and proteoglycans were also among them. These results demonstrated substantially enriched biological KEGG pathways implicated in OS (Figure 4D–F).

### 3.4. Random Forest Tree Screening

**Figure 5:**
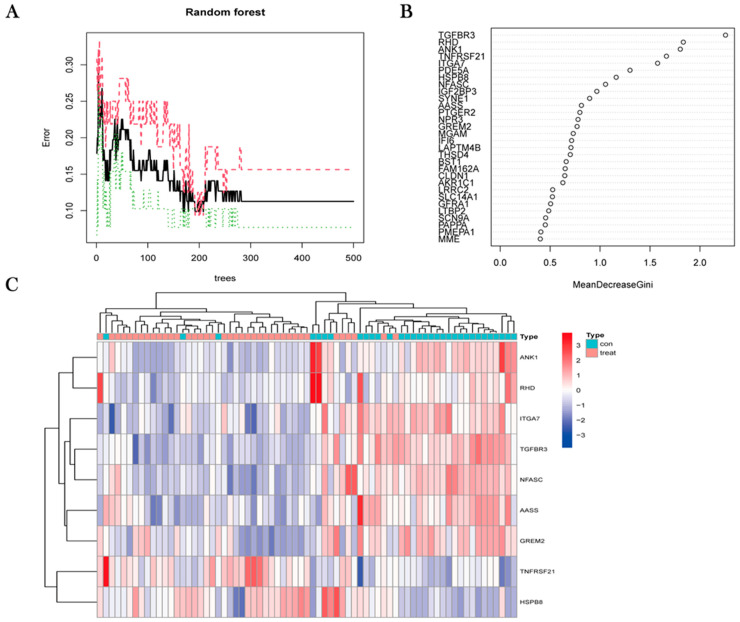
(**A**) Representative genes were identified by random forest. The total number of trees is represented on the *x*-axis, and the rate of error is represented on the *y*-axis. (**B**) The top 30 representative genes of importance were identified according to the random forest algorithm. (**C**) The unsupervised clustering heatmap shows the hierarchical clusters formed by the nine representative genes created by random forest in the combined dataset.

The 75 obtained DEGs were input into the random forest model, the cross-validation error was minimized to 169 trees, and the results were visualized (Figure 5A). Subsequently, nine representative genes with important points larger than one were identified by random forest. The importance of the DEGs is shown in Figure 5B. Among the nine representative genes, TNFRSF21 and HSPB8 were downregulated in the normal group and upregulated in the OS group. ANK1, RHD, ITGA7, TGFBR3, NFASC, AASS, and GREM2 were upregulated in the normal group and downregulated in the OS group (Figure 5C).

### 3.5. Construction and Validation of the Osteosarcoma-Related Gene Diagnostic Model

With the R package, a diagnostic prediction model for nine feature genes was successfully constructed, and the model was divided into three parts: the input layer, the hidden layer and the output layer. The principle of R package construction is to build a neural network model based on representative gene weights using a deep machine learning algorithm. The formula is as follows: Neural OS = ∑(Gene Score×Gene Weight). The accuracy of the model in predicting OS in the training and validation groups is shown in Table 2 and Table 3.

**Figure 6:**
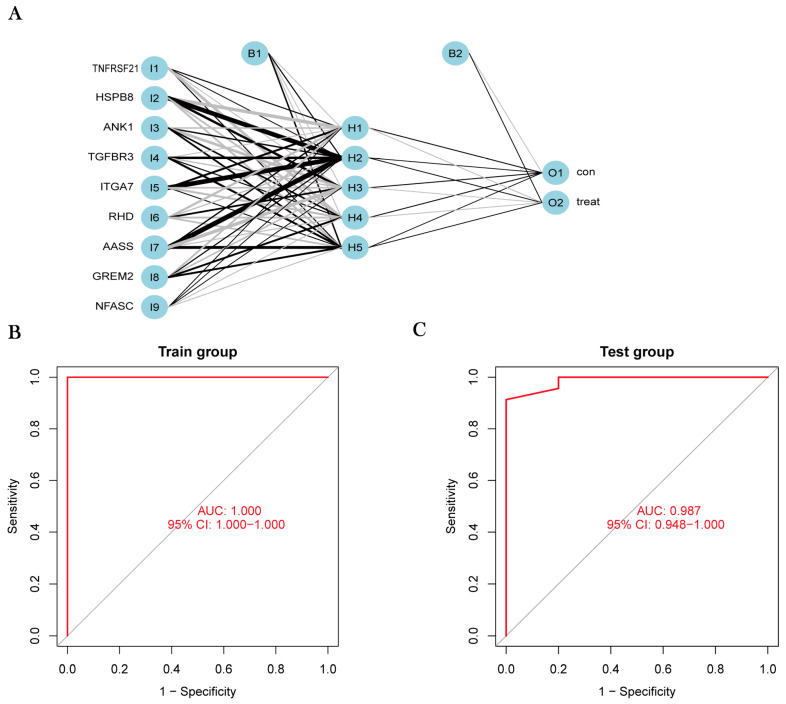
(**A**) Visualization results of the artificial neural network we constructed. (**B**) AUC of the training group after verifying the model. (**C**) AUC of the validation group after verifying the model.

The AUC of the diagnostic model constructed by representative genes was one [95% confidence interval (CI): 1–1], which indicates that the model has perfect diagnostic performance for OS. For the validation set, we used the same method to identify nine DEGs, which are the same as the training group. The validation group had an AUC of 0.987 [95% confidence interval (CI): 0.948–1], which greatly demonstrated the credibility and stability of the osteosarcoma-related gene diagnostic model (Figure 6C).

### 3.6. Immune Infiltration Analysis

**Figure 7:**
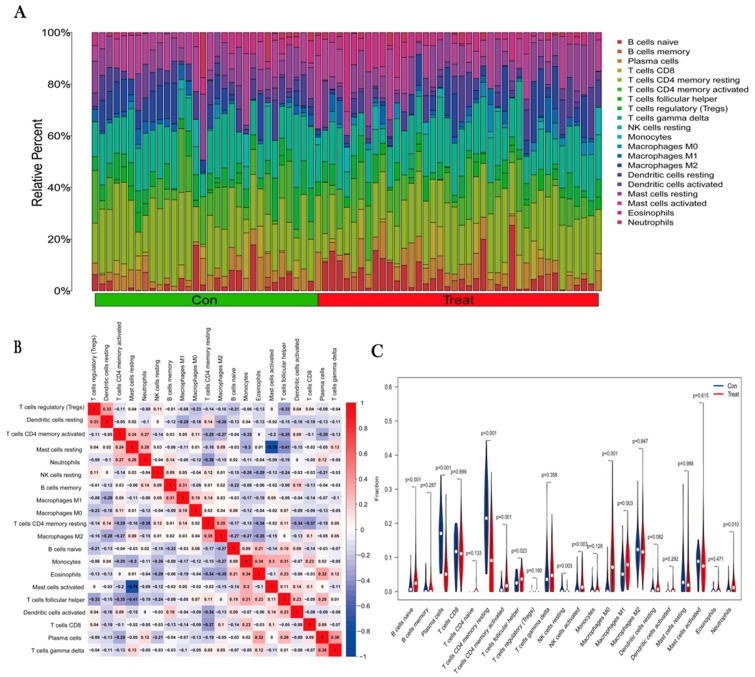
Immune components in tissues were estimated using CIBERSORT. (**A**) Proportions of 22 immune cell types in OS tissue (Treat) and normal paraneoplastic tissue (Con). (**B**) Correlation heatmap of 22 immune cell species. (**C**) Differences in the amount of immune cell infiltration between OS and normal group samples.

By performing immune infiltration analysis, we evaluated the proportion of 22 immune cell species in the OS group and normal group, visualized using bar graphs (Figure 7A). Immune cell correlation heatmap analysis (Figure 7B) showed that gamma delta T cells had the most significant positive correlation with plasma cells (r = 0.39). Resting mast cells were negatively correlated with activated mast cells (r = −0.75). A violin plot showing the results of the analysis of immune cell differences between the two groups (Figure 7C) showed lower levels of naïve B cells and M0 macrophages in normal paraneoplastic tissues than in osteosarcoma tissues, while the levels of regulatory T cells (Tregs) were higher than in osteosarcoma tissues (*p* < 0.05). The results of these studies showed some differences between the OS and normal groups in terms of immune cell content.

### 3.7. Cox Proportional Hazards Model and Survival Analysis

**Figure 8:**
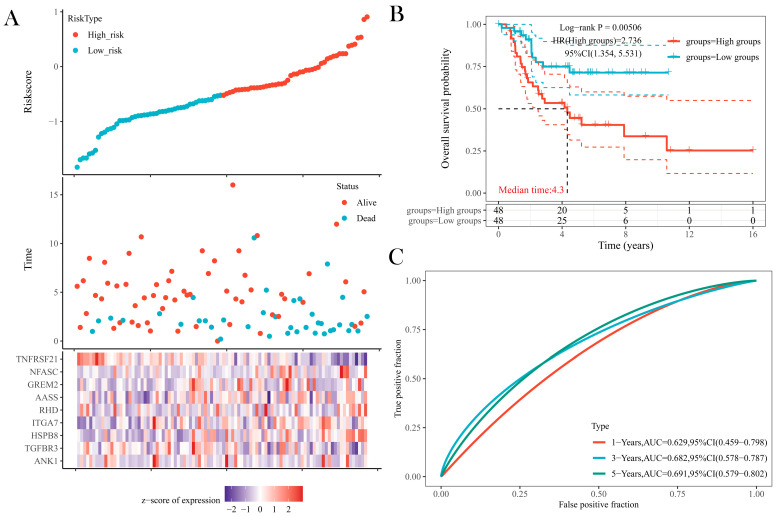
(**A**) Risk curves, survival status charts and risk heatmap for patients with osteosarcoma. The horizontal coordinates are risk values, and the vertical coordinates are risk scores, patient survival time (years) and nine representative genes. (**B**) The graph examines the effect of high- and low-risk groups on patient prognosis. The survival curve has the horizontal axis of the observation time and the vertical axis of the survival rate. (**C**) The ROC curve of this risk model at various times with AUC, where better AUC values indicate that the model has better predictive power.

Figure 8A shows a graphical representation of the risk curve, the survival status graph and the risk heatmap combined. They have the same horizontal coordinates and were all ordered from low to high based on the risk values of patients with osteosarcoma. In the risk curve, patients were divided into two groups of high and low risk, with higher risk scores indicating higher risk values. The survival status graph showed that the number of patient deaths gradually increased as the risk value increased. We can obtain from the heatmap that the expression of ANK1/TGFBR3/TNFRSF21 might be positively correlated with patient survival and that the expression of HSPB8/ITGA7/AASS might show a negative correlation. By Kaplan–Meier survival analysis (Figure 8B), the horizontal coordinate represented the survival time, and the subcolumn above the horizontal coordinate contained the number of patient-specific survival times for the two high- and low-risk groups. The vertical coordinate is the patient survival rate. It was obvious that patient survival decreased over time, with a *p*-value < 0.05 indicating a statistically significant difference between the high- and low-risk groups. The prognostic model constructed from representative genes predicted survival for 1, 3, and 5 years with AUCs of 0.629, 0.682, and 0.691, respectively. This indicated that the model performed well in predicting the survival prognosis of patients with osteosarcoma (Figure 8C). The distribution of copy number variations is shown in Figure S1. In addition, a heatmap was used to illustrate the copy number variations of the first 19 fragments and the fragment in which TGFBR3 is located (Figure S2). The important copy number variation genes in the osteosarcoma genome are listed in Supplementary Tables S1 and S2.

## 4. Discussion

An increasing number of studies based on public databases (GEO and TARGET) have been completed to characterize the biology of osteosarcoma. For example, Liang et al. identified key genes for osteosarcoma metastasis by analyzing the database and constructed a prognostic nomogram prediction model with gene scores [20]. Recently, some investigators have studied genes for the diagnosis of osteosarcoma. Sittiju et al. quantified the expression of five candidate genes as well as ezrin and vimentin by quantitative real-time polymerase chain reaction. Then, they analyzed them using mathematical models to efficiently differentiate OS from normal tissues, resulting in the selection of three candidate genes as a tool for OS detection and a tool to predict disease progression [21]. However, no investigators have used the screened signature genes to construct a diagnostic model for osteosarcoma. To the best of our knowledge, no studies have been conducted to develop diagnostic models for osteosarcoma using biomarkers as predictors. Our research was the first known study that used random forest analysis combined with ANN to construct a predictive model for OS genetic diagnosis, which performed well in distinguishing the OS group from the normal groups. The random forest algorithm has been successfully used as an integrated learning method to predict and identify clinical diseases with high efficiency and accuracy [22]. In medicine, ANNs have been used for the diagnosis, staging and recurrence prediction of prostate cancer since the 1990s [17]. Yang et al. also successfully proposed a Crohn’s disease diagnostic model using a similar method [8]. Therefore, these deep machine-learning algorithms are also applicable to the diagnosis of osteosarcoma. Diagnostic models constructed by using random forests and artificial neural networks have good accuracy and precision.

In this study, we screened 75 DEGs related to OS using three datasets and identified nine representative genes by random forest analysis. Enrichment analysis of DEGs by GO and KEGG indicated a link between cellular processes such as ECM, extracellular structure organization, ECM-receptor interaction, PI3K-Akt signaling pathway, TGF-beta signaling pathway and OS occurrence, which has been previously reported [23,24].

In our research, TNFRSF21 was the most representative gene identified by the random forest classifier. TNFRSF21 is a member of the tumor necrosis factor receptor superfamily that possesses an intracellular death domain [25]. Studies have shown that TNFRSF21 is expressed at elevated levels in different tumor tissues, including prostate, breast and ovarian cancers [26]. One study found that eIF3b silencing led to the upregulation of TNFRSF21, which in turn induced cell death in U2OS cells [27]. HSPB8 is a member of the HSPB family. It has been reported that it may be involved in the regulation of cell proliferation and apoptosis, and even carcinogenesis, mainly due to the chaperoning role of binding to Bag3 (a macrophage stimulator) [28,29]. Regarding the downregulated gene ANK1, recent studies have shown that the CpG island in the ANK1 host gene promoter of mir-486 is highly methylated in osteosarcoma cell lines [30]. This result suggested that this is a common epigenetic regulation in osteosarcoma cell lines. TGFBR3, which encodes a receptor that is a membrane proteoglycan, is the most highly expressed TGFBR without kinase activity. However, it binds to all three isoforms of TGF-β and promotes the ligand of ligands to TGFBR2, especially TGF-β2 [31]. The RNA-seq results of Xie et al. showed that TGFBR3 expression was downregulated during OS tumorigenesis and upregulated during OS lung metastasis [32]. Integrin α7 (ITGA7), encoding a product belonging to the integrin alpha chain family, includes extracellular matrix (ECM)-binding protein [33]. Laminin-1 is a basement membrane protein on the surface of skeletal muscle cells and myofibers, and ITGA7 can act as a receptor for laminin-1 [34]. GREM2, which belongs to the DAN family, regulates tumor development by regulating bone morphogenetic proteins and making them interact with each other [35].

HSPB8/RHD/AASS/NFASC has not yet been found to be associated with osteosarcoma. Related studies suggest that although the molecular mechanism of HSPB8 in cancer requires deeper investigation, HSPB8 may be a significant factor in the initiation and progression of tumors and may be a biomarker for the treatment of certain types of tumors [36]. To date, there are no studies combining HSPB8 and human osteosarcoma. AASS encodes α-aminoadipic acid hemi-aldol synthase, which is involved in the catabolism of lysine. NFASC encodes a transmembrane protein. It plays an important role in nervous system development and Ranvier functional nodes [37]. By immune infiltration analysis, significant differences could be found between tumor tissue and normal tissue, mainly in macrophages, natural killer (NK) cells and B lymphocytes. It has been shown that the development of osteosarcoma is closely related to NK cell and macrophage infiltration [38,39]. We further found by survival analysis of nine representative genes that they could successfully construct risk models to predict the prognosis (survival time) of patients with osteosarcoma. Additionally, copy number variants suggest that TGFBR3 may be more representative.

The clinicopathological biopsy is the diagnostic criterion for osteosarcoma, and only one fine-needle aspiration biopsy is sufficient for a definitive diagnosis. It has been reported in the literature that fine needle aspiration biopsies were performed in 59 adult patients with soft tissue sarcoma who underwent final surgery without removal of these biopsy passages, but there was no increase in local recurrence of the tumor relative to reported studies in which biopsy passages were removed at the time of final surgery [40]. Most patients opt for closed biopsy for the first time, but in cases where a definitive diagnosis is not made at one time or where the tissue entity does not match the radiographic diagnosis, open biopsy is now the only option clinically available again. Although open biopsy is the most reliable diagnostic technique, it may elevate the risk of complications such as medically induced vascular and nerve injury, wound nonhealing, wound infection, tumor contamination of the biopsy access and local recurrence. The construction of the present model can address this clinical problem of fine needle aspiration biopsy failure to some extent.

Despite the success of this study in establishing the first diagnostic model for osteosarcoma based on a public database, there should still be some limitations. First, because a public database was used, there were a small number of mismatches between OS patients and controls. Because of the consideration of data matching, the sample size incorporated into the training group is relatively small. Second, our training cohort consisted of different sequencing platform datasets. Although corrections between different data platforms were used, there still existed potential errors, which, to some extent, could affect the study results. In the future, studies with large amounts of matched data will be needed to demonstrate the reliability of this finding. Third, due to the lack of experimental validation, the newly identified potential markers need further consideration. Overall, we provide a new horizon for the early accurate diagnosis of OS and offer potential biomarkers that can be referenced for precise treatment.

## 5. Conclusions

Nine potential osteosarcoma biomarkers were obtained through this study, and a random forest combined with an artificial neural network model for osteosarcoma diagnosis was successfully constructed and validated. In turn, it provides a helpful basis for early screening of OS and promotes further research on OS diagnosis. In conclusion, our study has implications for the early diagnosis of OS.

## Figures and Tables

**Table 1:** The basic information of the Training group and Validation group.

DataSets Series	Platform	Tumor	Normal	Group
GSE14359	GPL96	10	2	Training group
GSE99671	GPL20148	18	18	Training group
GSE126209	GPL20301	11	12	Training group
GSE19276	GPL6848	23	5	Validation group

**Table 2:** The accuracy of the model in the training group.

Group	Normal	OS	Total
Normal	32	0	32
OS	0	39	39

**Table 3:** The accuracy of this model in the validation group.

Group	Normal	OS	Total
Normal	5	0	5
OS	4	19	23

## Data Availability

All data for this study were obtained by downloading through the Gene Expression Omnibus and Therapeutically Applicable Research to Generate Effective Treatments, as described in the methods section of this paper. If you have any questions, please contact the authors, and we will provide them.

## Supplementary Materials

The following supporting information can be downloaded at https://www.mdpi.com/article/10.3390/jpm13030447/s1: Figure S1: Distribution of genomic amplification/deletion variation fragments in patients with osteosarcoma; Figure S2: Copy number variation heatmap of the first 19 fragments and the fragment where TGFBR3 is located; Table S1: The identified peak regions with copy number amplification and the genes involved in the regions; Table S2: The identified peak regions with copy number deletions and the genes involved in the regions. Table S3: Identified copy number amplification and deletion peak regions.

## Abbreviations

The following abbreviations are used in this article and may be helpful to read:

AASS	Aminoadipate Semialdehyde Synthase
ANK1	Ankyrin 1
ANN	Artificial neural network
AUC	Area Under Curves
DEG	differentially expressed gene
ECM	Extracellular matrix
GEO	Gene Expression Omnibus
GO	Gene Ontology
GREM2	Gremlin 2, DAN Family BMP Antagonist
HSPB8	Heat Shock Protein Family B (Small) Member 8
ITGA7	Integrin Subunit Alpha 7
KEGG	Kyoto Encyclopedia of Genes
MRI	Magnetic Resonance Imaging
NFASC	Neurofascin
NK	natural killer
OS	Osteosarcoma
RHD	Rh Blood Group D Antigen
ROC	receiver operating characteristic
TARGET	Therapeutically Applicable Research To Generate Effective Treatments
TGFBR3	Transforming Growth Factor Beta Receptor 3
TNFRSF21	TNF Receptor Superfamily Member 21
