# Supplementary figures and images for: Construction of Osteosarcoma Diagnosis Model by Random Forest and Artificial Neural Network
Source: J Pers Med. 2023 Feb 28;13(3):447. doi: 10.3390/jpm13030447 (PMC10056981; doi:10.3390/jpm13030447)

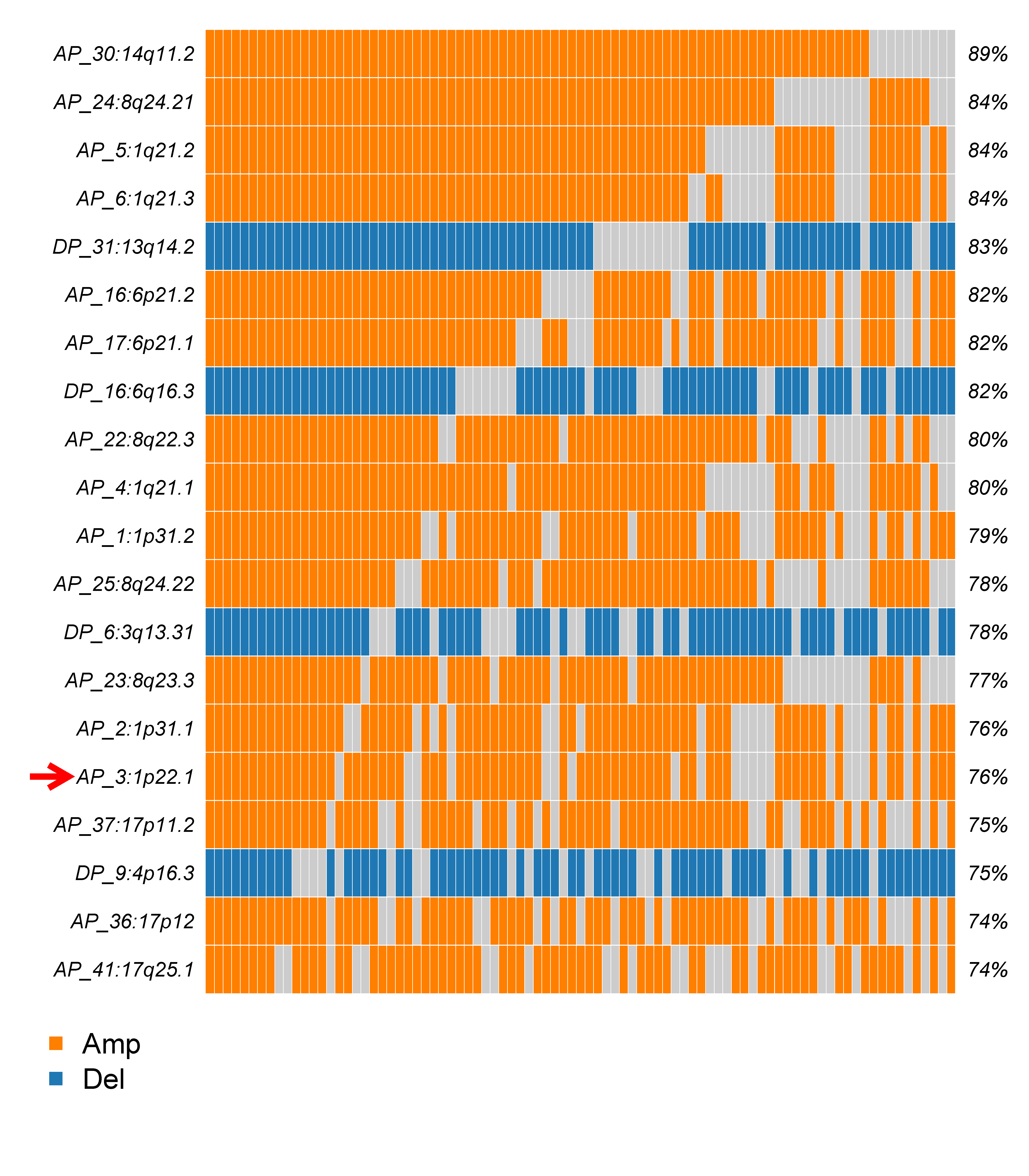

Supplement: Supplementary file 1 [file jpm-13-00447-s001.zip › Figure S2.tiff]
